# RIG-I Mediates the Co-Induction of Tumor Necrosis Factor and Type I Interferon Elicited by Myxoma Virus in Primary Human Macrophages

**DOI:** 10.1371/journal.ppat.1000099

**Published:** 2008-07-11

**Authors:** Fuan Wang, Xiujuan Gao, John W. Barrett, Qing Shao, Eric Bartee, Mohamed R. Mohamed, Masmudur Rahman, Steve Werden, Timothy Irvine, Jingxin Cao, Gregory A. Dekaban, Grant McFadden

**Affiliations:** 1 BioTherapeutics Research Group, Robarts Research Institute, London, Ontario, Canada; 2 Department of Microbiology and Immunology, The University of Western Ontario, London, Ontario, Canada; 3 Department of Anatomy & Cell Biology, The University of Western Ontario, London, Ontario, Canada; 4 Department of Molecular Genetics and Microbiology, College of Medicine, University of Florida, Gainesville, Florida, United States of America; 5 National Microbiology Laboratory, Public Health Agency of Canada, Winnipeg, Manitoba, Canada; University of Washington, United States of America

## Abstract

The sensing of pathogen infection and subsequent triggering of innate immunity are key to controlling zoonotic infections. Myxoma virus (MV) is a cytoplasmic DNA poxvirus that in nature infects only rabbits. Our previous studies have shown that MV infection of primary mouse cells is restricted by virus-induced type I interferon (IFN). However, little is known about the innate sensor(s) involved in activating signaling pathways leading to cellular defense responses in primary human immune cells. Here, we show that the complete restriction of MV infection in the primary human fibroblasts requires both tumor necrosis factor (TNF) and type I IFN. We also demonstrate that MV infection of primary human macrophages (pHMs) activates the cytoplasmic RNA sensor called retinoic acid inducible gene I (RIG-I), which coordinately induces the production of both TNF and type I IFN. Of note, RIG-I sensing of MV infection in pHMs initiates a sustained TNF induction through the sequential involvement of the downstream IFN-regulatory factors 3 and 7 (IRF3 and IRF7). Thus, RIG-I-mediated co-induction of TNF and type I IFN by virus-infected pHMs represents a novel innate defense mechanism to restrict viral infection in human cells. These results also reveal a new regulatory mechanism for TNF induction following viral infection.

## Author Summary

Myxoma virus, a member of the cytoplasmic DNA poxvirus family, causes a lethal disease called myxomatosis in the European rabbit. It has been known for over a half-century that other vertebrates, including humans, are highly resistant to myxoma virus infection, even following direct injections of live myxoma virus. Interestingly, myxoma virus has been recently shown to potently infect and kill malignant human tumors xenografted in mice. However, little is known about how normal human immune cells sense and suppress myxoma virus multiplication at the cellular level. Here, we show that the cytoplasmic RNA helicase RIG-I is the major sensor that detects invading myxoma virus in primary human macrophages and triggers the co-induction of both tumor necrosis factor and type I interferon. Together, tumor necrosis factor and type I interferon inhibit myxoma virus in otherwise permissive cells, such as human fibroblasts. Conceptually, our study thus demonstrates that intracellular RNA sensors may, in general, play a more important role than previously thought in the innate antiviral responses against DNA virus infections in human cells.

## Introduction

Myxoma virus (MV), a member of the poxvirus family, is a large cytoplasmic DNA virus that infects only rabbits [1],[2]. No other vertebrate species outside of lagomorphs, including humans, have ever been reported to contract a productive MV infection. This strict host specificity of MV provides an important avenue to study how cross-species virus infections can be manipulated, and how zoonotic infections might be regulated in humans. For instance, the mitogen-activated protein kinase (MAPK) Erk1/2-type I interferon (IFN-α/β)-STAT1 signaling cascade has been revealed as the principal antiviral defense pathway which restricts MV infection in primary mouse embryo fibroblasts. In fact, disruption of the STAT1 signaling cascade renders normally resistant mice highly susceptible to lethal MV infection [3]. These observations demonstrate the importance of defining the functional antiviral mechanisms in primary cells, as compared to transformed or immortalized cell lines [4]. Little is known, however, about the innate defense pathways that inhibit MV infection in normal primary human cells.

Innate cellular defenses can be triggered by a variety of mechanisms, including host recognition of pathogen-associated molecular patterns (PAMPs) through pattern recognition receptors, such as Toll-like receptors (TLRs) and cytoplasmic nucleic acid sensors [5],[6]. Although TLR3, TLR7, TLR8 and TLR9 are known to recognize certain viral nucleic acids, these TLRs are exclusively localized in the endosomal compartments and therefore might be unable to sense cytoplasmic viral PAMPs [7].

Cellular surveillance of invading DNA viruses likely involves multiple viral PAMPs, particularly certain atypical RNAs. For example, many DNA viruses produce dsRNA and/or 5′-triphosphate RNA, as viral by-products, during their replicative life cycles [5],[8],[9]. Both viral dsRNA and 5′-triphosphate RNA can potently activate the cytoplasmic RNA sensor called retinoic acid inducible gene I (RIG-I) [9]–[12]. In contrast, certain other viral RNA moities are specifically detected by another cytoplasmic RNA sensor called melanoma differentiation-associated gene 5 (MDA5) [13]. Thus, it is highly plausible that infecting DNA viruses may generate various “accidental” RNA ligands which engage cytoplasmic RNA sensors. Current reported studies are focusing predominantly on the roles of RIG-I and MDA5 in triggering innate defenses against RNA viruses [13],[14]. Consequently, it remains to be determined how critical RIG-I and MDA5 might be in sensing DNA viruses.

Host recognition of viral infection normally activates multiple evolutionarily conserved signaling pathways, such as NF-κB and the IFN-regulatory factors 3 and 7 (IRF3 and IRF7) [15]. Activation of these pathways often culminates in the induction of an array of antiviral cytokines, including type I IFN and tumor necrosis factor (TNF), which are widely considered crucial components of innate antiviral immunity [16]–[18]. However, although the signaling cascades linking RIG-I and MDA5 sensing to IFN-α/β induction have been extensively characterized [5],[9],[19], so far little is known as to how RIG-I and/or MDA5 signaling may be linked with TNF expression.

Here, we show that MV is permissive in cultured primary human fibroblasts and that potent restriction of MV infection in primary human fibroblasts requires TNF in conjunction with type I IFN. In contrast, MV infection of primary human macrophages (pHMs) triggers induction of both TNF and type I IFN that prevents permissive infection. Furthermore, we show that MV infection of pHMs is sensed largely by RIG-I, and the subsequent TNF induction is mediated through the sequential involvement of IRF3 and IRF7. Our results demonstrate that RIG-I-mediated co-induction of TNF and IFN is a novel innate defense mechanism that restricts MV proliferation in normal primary human cells and reveals a hitherto unrecognized regulatory pathway for TNF induction in virus-infected cells.

## Results

### TNF and type I IFN are required for complete restriction of MV infection in primary human fibroblasts

**Figure 1:**
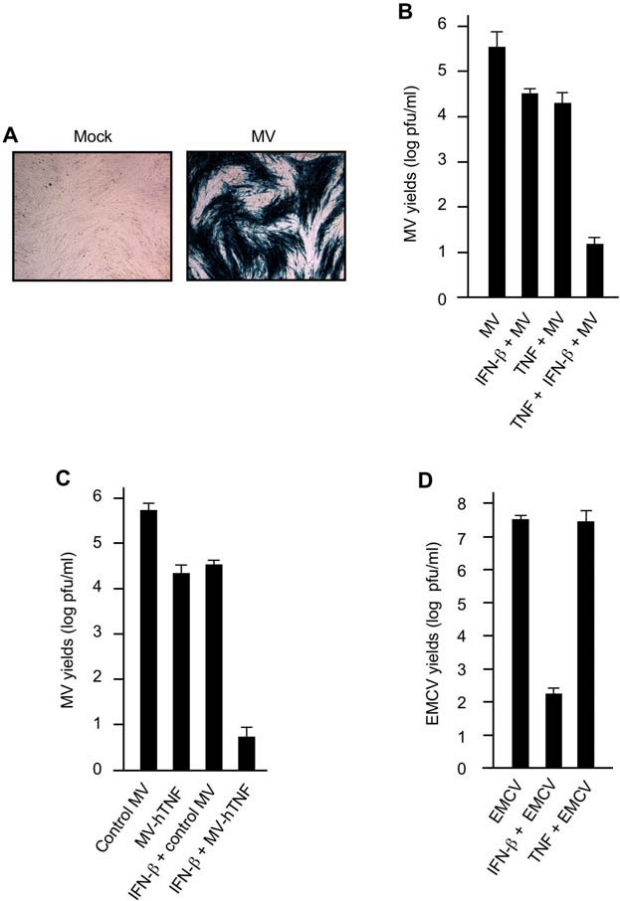
TNF and IFN-β are required for potent restriction of MV infection in primary human fibroblasts. (A) Primary human skin fibroblasts CCD-922Sk were mock-infected or infected with MV. X-gal staining was performed to visualize blue MV foci 48 h after infection. (B) CCD-922Sk fibroblasts were infected with MV in the absence or presence of TNF or IFN-β alone or TNF plus IFN-β. MV yields were determined by the standard plaque assay using BGMK cells at 48 h after infection. (C) CCD-922Sk fibroblasts were infected with the control MV (constructed with the same vector but no human TNF gene) or the recombinant MV expressing human TNF (MV-hTNF) in the absence or presence of IFN-β. The viral yields of both control MV and MV-hTNF were determined by the standard plaque assay using RK-13 cells at 48 h after infection. (D) CCD-922Sk fibroblasts were infected with EMCV in the absence or presence of IFN-β or TNF. EMCV quantities were titrated by the standard plaque assay using BHK cells at 48 h after infection. Data in (B), (C) and (D) represent mean +/− SD.

The potent oncolytic ability of MV in treating human tumors in mouse xenograft models [20]–[22] prompted our interest in studying MV infectivity in normal primary human cells. Therefore, we initially infected CCD-922Sk primary human skin fibroblasts with a MV construct expressing β-galactosidase (β-gal) under the control of a late viral promoter. Following low multiplicity of infection, X-gal staining revealed that, in contrast to the nonpermissive phenotype in MV-infected primary murine cells [3], MV formed classic productive blue foci (Figure 1A), indicating that CCD-922Sk fibroblasts were permissive for MV infection. Similar results were obtained with a variety of other primary human fibroblasts tested in this fashion (data not shown).

Next, we infected CCD-922Sk fibroblasts with MV in the presence of exogenous human IFN-β as a prototypic representative of type I IFN to ascertain whether this cytokine could reverse the permissive phenotype [18],[23]. As shown in Figure 1B, exogenous IFN-β reduced MV yield by approximately one log. To characterize other potential host factors that might also inhibit MV infection in human fibroblasts, we then investigated the potential anti-MV effects of other antiviral cytokines. For example, TNF has been shown to possess antiviral activities under certain circumstances, particularly in collaboration with type I IFN [24]. To this end, we infected CCD-922Sk fibroblasts with MV in the presence of TNF alone, or together with IFN-β. We observed that approximately one log reduction in MV yield was obtained with TNF treatment alone (Figure 1B). Interestingly, this anti-MV effect of either cytokine alone was considerably enhanced when the two cytokines were combined, as that treatment inhibited MV replication by more than 4-log (Figure 1B). To further validate the anti-MV properties of TNF, we then constructed a recombinant MV that constitutively expresses human TNF (MV-hTNF, Figure S1). Compared with the control MV, MV-hTNF yield was approximately a log lower in CCD-922Sk fibroblasts (Figure 1C). Of note, this reduction could be reversed by a TNF neutralizing antibody (data not shown). Significantly, the replication of MV-hTNF was now nearly abolished by IFN-β alone (Figure 1C). We further observed that, although the relative anti-MV potencies of TNF and type I IFN varied between individual infections, TNF in combination with either IFN-β or IFN-αA uniformly restricted MV replication in all primary human cells tested (data not shown). Taken in aggregate, these data demonstrate that TNF synergizes with type I IFN to achieve potent innate restriction of MV replication in primary human fibroblasts.

To further characterize the antiviral potencies of IFN-β and TNF in CCD-922Sk fibroblasts, we then infected the cells with encephalomyocarditis virus (EMCV), an RNA virus from the picornaviridae family, in the presence of IFN-β or TNF. Unlike MV, EMCV yield could be inhibited by more than 5-log in CCD-922Sk fibroblasts by IFN-β alone (Figure 1D). However, TNF alone exhibited no anti-EMCV efficacy (Figure 1D), nor did it affect IFN-β inhibition of EMCV (data not shown). Together, these results indicate that, at least in human fibroblasts, whether TNF is needed to synergize with type I IFN to achieve the full antiviral state depends significantly upon the infecting virus.

### MV infection elicits robust induction of TNF and type I IFN in primary human macrophages

**Figure 2:**
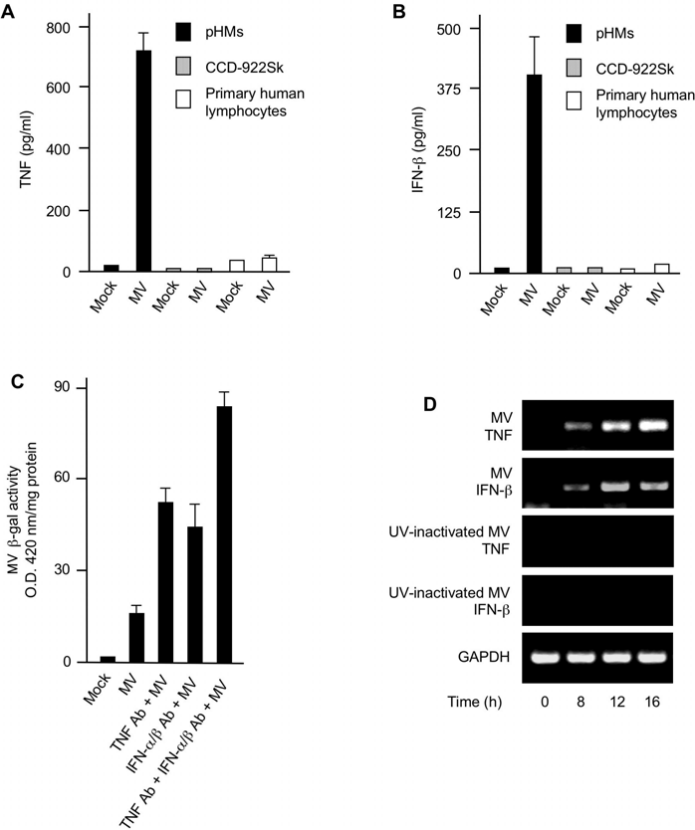
Primary human macrophages produce TNF and IFN-β in response to MV infection. (A, B) Primary human macrophages (pHMs), CCD-922Sk fibroblasts and primary human lymphocytes were mock-infected or infected with MV. TNF (A) and IFN-β (B) accumulation in the culture supernatants was assessed by ELISA 24 h post-infection. (C) pHMs were mock-infected or infected with MV in the absence or presence of TNF neutralizing antibody (Ab) or IFN-α/β neutralizing Ab alone or TNF Ab plus IFN-α/β Ab as specified. MV β-gal activity was determined by measuring absorbance at 420 nm and normalized to the total cellular protein levels at 48 h after infection. (D) pHMs were infected with MV or UV-inactivated MV for various times (below lanes) and total RNA was analyzed by RT-PCR for induction of TNF and IFN-β mRNA. GAPDH was used as control. Data in (A), (B) and (C) represent mean +/− SD.

The involvement of TNF and type I IFN in restricting MV infection prompted us to explore macrophages as a model system to investigate the cytokine inducibility by MV infection because macrophages are generally considered as particularly potent TNF producers in response to a variety of stimuli [25]. To this end, we infected monocyte-derived pHMs with MV and then measured TNF production in response to the viral infection. A significant increase in secreted TNF protein was detected in the media of MV-infected pHMs, but not in the media of MV-infected CCD-922Sk fibroblasts or primary human lymphocytes (Figure 2A). Because type I IFN was shown to potentiate the anti-MV effects of TNF (Figures 1B and C), we therefore examined whether MV infection could also induce type I IFN expression. In accord with TNF production, IFN-β was only detected in the media of MV-infected pHMs (Figure 2B). Next, we examined MV β-gal activity driven by a late viral promoter following MV infection in pHMs as an indicator for MV DNA replication status. As shown in Figure 2C, only a small amount of MV β-gal activity was detected in pHMs but this level could be increased with neutralizing antibody to either TNF or type I IFN alone, and further elevated by adding the two antibodies together. In total, these results indicate that MV-infected pHMs are restrictive because of the secreted TNF and type I IFN induced as a response to MV infection.

Although TNF synthesis and secretion is regulated at multiple levels, the transcriptional upregulation of TNF gene expression is a critical point of control over TNF production [26]. Therefore, we used RT-PCR to determine whether MV infection elicited the enhanced expression of TNF mRNA in pHMs. Consistent with the TNF protein data, MV infection resulted in a substantial increase in both TNF and IFN-β mRNA, with similar kinetics (Figure 2D). No appreciable amounts of TNF or IFN-β mRNAs were detected in either MV-infected CCD-922Sk fibroblasts or primary human lymphocytes (data not shown). Collectively, these results indicate that the co-induction of TNF and IFN-β secretion from MV-infected pHMs was regulated through augmented mRNA transcription of each cytokine.

Next, to determine whether MV replication was required to induce TNF or type I IFN expression in pHMs, we prepared UV-inactivated MV that was no longer able to synthesize significant levels of functional viral RNA or protein in a fashion similar to that reported for vaccinia virus [27]. Radioiodination labeling showed that the UV-inactivated MV particles retained the ability to enter into the cell interior (data not shown). As demonstrated in Figure 2D, UV-inactivated MV was unable to upregulate mRNA expression of either TNF (3rd panel) or IFN-β (4th panel) in pHMs. Together, these data suggest that the *de novo* synthesis of MV macromolecules is critical for triggering the cellular signaling cascades leading to TNF and IFN-β induction in pHMs.

### RIG-I mediates MV-elicited induction of TNF and type I IFN in primary human macrophages

**Figure 3:**
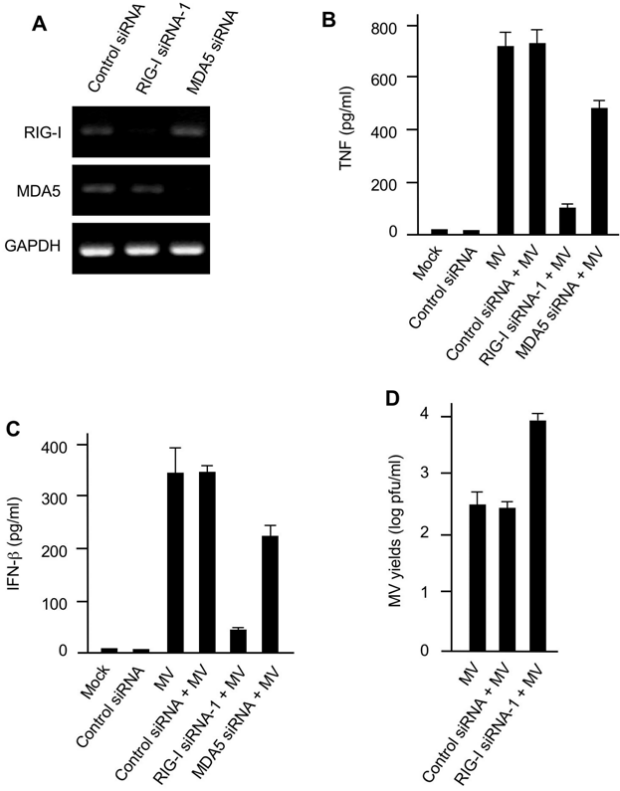
MV-elicited production of TNF and IFN-β is mediated by RIG-I in primary human macrophages. (A) pHMs were transfected with control siRNA or siRNAs targeting cytoplasmic RNA sensors RIG-I (top panel) or MDA5 (middle panel). The cells were analyzed 48 h later by RT-PCR for the indicated mRNA levels. GAPDH was used as control. (B, C) RIG-I is critically required for MV-elicited production of both TNF (B) and IFN-β (C) in pHMs. pHMs or various siRNA pHMs were mock-infected or infected with MV and the accumulation of TNF and IFN-β in the culture supernatants was assessed by ELISA 24 h post-infection. (D) RIG-I mediates cellular restriction to MV infection in pHMs. pHMs or control siRNA or RIG-I siRNA pHMs as indicated were infected with MV. MV yields were determined by the standard plaque assay using BGMK cells at 48 h after infection. Data in (B), (C) and (D) represent mean +/− SD.

Like all poxviruses, MV synthesizes its viral DNA, RNA and protein in the cytoplasm of infected cells [2]. Thus, the finding that MV-elicited TNF and IFN-β mRNA induction in pHMs required active MV infection (Figure 2D) led us to postulate that cytoplasmic sensors, such as RIG-I or MDA5 [12],[13], might sense cytoplasmic nucleic acid intermediates derived from MV replication. To test this hypothesis, we examined the basal levels of RIG-I and MDA5 in pHMs. As revealed by RT-PCR, mRNAs for the nucleic acid sensors RIG-I and MDA5 were constitutively expressed in pHMs (Figure 3A). Next, to investigate whether these sensors were involved in MV-elicited TNF or IFN induction, we employed siRNAs to individually suppress the expression of RIG-I and MDA5 in pHMs, as previous reports have shown that siRNA transfection could efficiently inhibit a variety of target genes in primary macrophages [28],[29]. We observed that transfection of RIG-I siRNA-1 and MDA5 siRNA significantly knocked down the mRNA expression of RIG-I (Figure 3A, top) and MDA5 (Figure 3A, middle). We then conducted MV infections in the pHMs in which the expression of RIG-I and MDA5 mRNA had been respectively silenced. As shown in Figure 3B, MDA5 mRNA suppression reduced MV-elicited TNF production only by approximately 30%, whereas RIG-I mRNA knockdown inhibited TNF induction by 85% in MV-infected pHMs (Figure 3B).

Because IFN-β was co-induced together with TNF in MV-infected pHMs (Figure 2), we then examined whether MV-elicited IFN-β expression was also mediated by RIG-I. As demonstrated in Figure 3C, RIG-I silencing reduced IFN-β production comparably to that of TNF. Importantly, virus yield analysis further revealed that RIG-I knockdown increased MV progeny production by 30-fold (Figure 3D) and augmented MV-derived β-gal activity by 80% (data not shown). Taken together, these data suggest that RIG-I is a major common mediator for MV-elicited induction of TNF and type I IFN in infected pHMs and that knockdown of RIG-I reduces this co-induction and thereby renders pHMs permissive for MV infection.

To verify the specificity of RIG-I siRNA silencing, we employed an additional RIG-I siRNA designated RIG-I siRNA-2. As shown in Figure S2A, RIG-I siRNA-2 displayed a silencing potency indistinguishable from that of RIG-I siRNA-1. Phenotypically, RIG-I silencing by RIG-I siRNA-2 reduced the MV-induced expressions of both TNF and IFN-β to the similar levels as seen with RIG-I siRNA-1 (Figures S2B and S2C). Because the sequences of RIG-I siRNA-1 and RIG-I siRNA-2 are different, it is thus unlikely that these siRNA oligos both knockdown a common off-target gene that is responsible for MV-elicited expression of TNF and type I IFN. Moreover, we also observed that RIG-I siRNAs used here themselves did not trigger undesired type I IFN response in pHMs (Figures S3A and S3B) [30],[31].

Next, to examine for any potential role of TLRs in MV-infected pHMs, we silenced the expressions of TLR4, MyD88 and Trif using specific siRNAs. These were chosen because all known TLRs use either the adaptor MyD88 or Trif to transduce signals, except for TLR4, which uses both adaptors MyD88 and Trif [7]. We observed that inhibition of TLR signaling with any of these siRNAs had no influence on either TNF or type I IFN production in MV-infected pHMs (data not shown).

### IRF3 is essential for RIG-I-mediated induction of TNF and type I IFN in primary human macrophages

**Figure 4:**
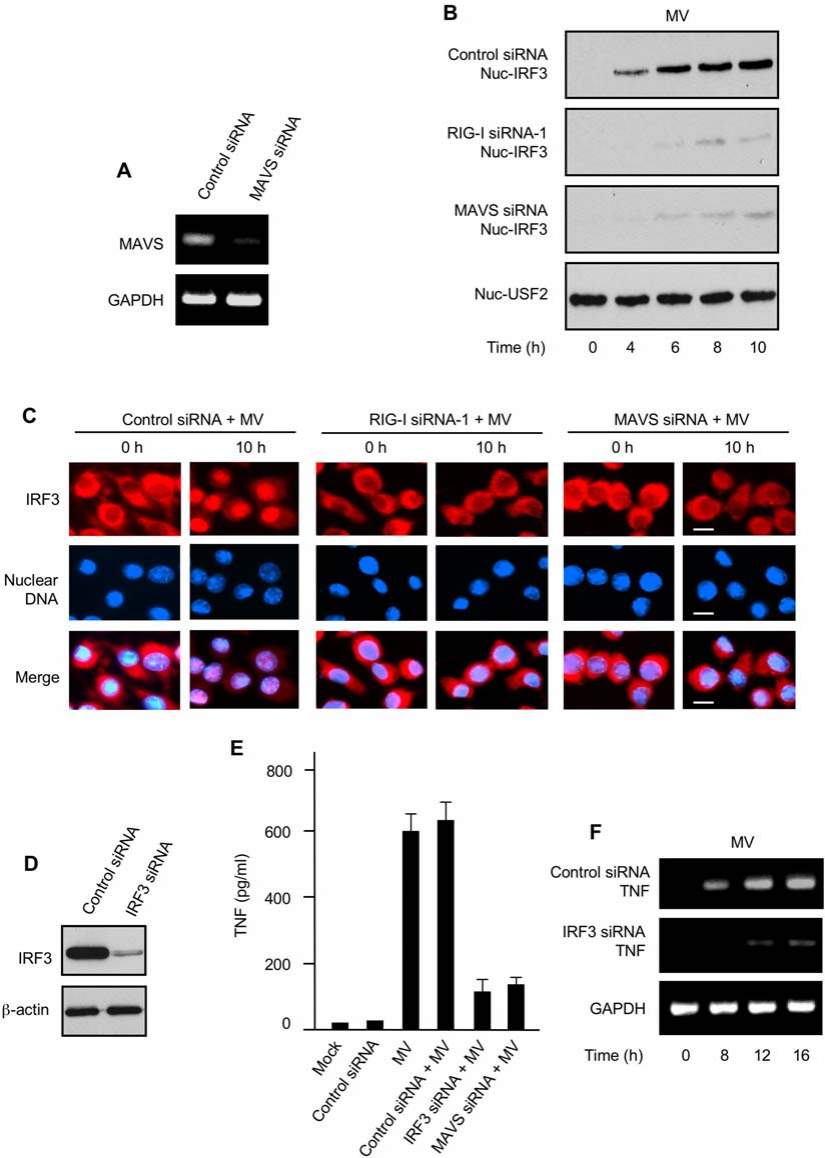
IRF3 is critical for triggering MV-elicited TNF induction in primary human macrophages. (A) pHMs were transfected with control siRNA or MAVS siRNA. The cells were analyzed 48 h later by RT-PCR for the indicated mRNA levels. GAPDH was used as the control. (B) Control siRNA pHMs or RIG-I siRNA or MAVS siRNA pHMs as indicated were infected with MV for various times (below lanes) and the nuclear extracts were probed for nuclear IRF3 (nuc-IRF3). Nuclear USF2 (nuc-USF2) was used as nuclear protein loading control. (C) Control siRNA pHMs or RIG-I siRNA or MAVS siRNA pHMs as indicated were infected with MV for 0 h or 10 h and were immunofluorescence-stained for IRF3 (red). Nuclear DNA was counterstained with DAPI (blue). Bars are 10 µm. (D) pHMs were transfected with control siRNA or IRF3 siRNA as indicated and were analyzed 72 h later by immunoblotting for IRF3 protein levels. β-actin was used as control. (E) pHMs or various siRNA pHMs as indicated were infected with MV for 24 h and TNF protein in the culture supernatants was assessed by ELISA. Data represent mean +/− SD. (F) Control siRNA pHMs or IRF3 siRNA pHMs were infected with MV for various times (below lanes) and the cells were analyzed by RT-PCR for the indicated TNF mRNA levels. GAPDH was used as control.

Upon ligand engagement, RIG-I recruits the adaptor protein MAVS [32], also known as VISA, IPS-1 or Cardif [33]–[35], resulting in the downstream activation and nuclear localization of IRF3, a central transcription factor for IFN-α/β expression [36],[37]. Importantly, recent studies demonstrate that IRF3 is also important for TNF expression [38]–[41]. To investigate whether signaling from MAVS to IRF3 is critically involved in RIG-I-mediated induction of TNF or type I IFN following MV infection, we first silenced MAVS expression in pHMs using MAVS siRNA (Figure 4A). We then prepared pHM nuclear extracts and examined, by immunoblotting, IRF3 nuclear translocation with or without MAVS silencing in comparison with RIG-I silencing. As revealed in Figure 4B (top panel), MV infection robustly induced IRF3 nuclear translocation [36]. This nuclear mobilization of IRF3 was strongly inhibited by knockdown of either RIG-I (Figure 4B, 2nd panel) or MAVS (Figure 4B, 3rd panel). Immunofluorescent staining revealed a similar distribution trend for IRF3 following MV infection (Figure 4C). Together, these results show that MV-induced IRF3 activation is dependent on the functional integrity of upstream signaling of both RIG-I and MAVS.

To investigate the physiological significance of MV-induced IRF3 activation in pHMs, we employed IRF3 siRNA to specifically knock down IRF3 expression (Figure 4D). This knockdown of IRF3 substantially reduced TNF protein secretion in the supernatant of MV-infected pHMs (Figure 4E). Similarly, MAVS silencing also strongly inhibited MV-elicited TNF production in pHMs (Figure 4E). These results suggest that MAVS-IRF3 signaling is important for TNF production in MV-infected pHMs. Consistent with these TNF protein data, we also observed that induction of MV-elicited TNF and IFN-β mRNA were both inhibited by IRF3 silencing (Figure 4F and data not shown), thus indicating that IRF3 regulates TNF and IFN-β production mainly through transcriptional activation. Taken together, our findings suggest that the RIG-I-MAVS-IRF3 signaling axis, a canonical cascade that is involved in IFN-α/β induction following RNA virus infections, can also be exploited in pHMs to co-transmit TNF-inducing signals emanating from a cytoplasmic DNA virus infection.

### IRF7 is required for sustaining RIG-I-mediated TNF induction in primary human macrophages

**Figure 5:**
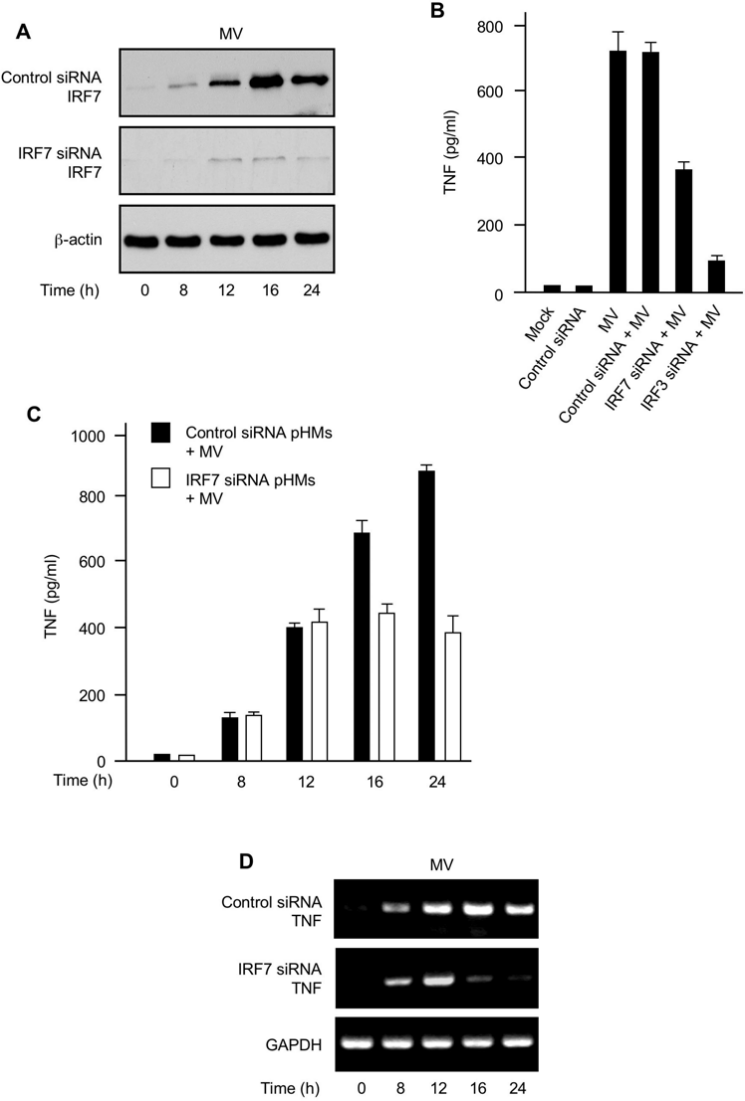
IRF7 is crucial for sustaining MV-elicited TNF induction in primary human macrophages. (A) Control siRNA pHMs or IRF7 siRNA pHMs were infected with MV for various times (below lanes) and the whole cell lysates were analyzed for MV-induced expression of IRF7 protein. β-actin was used as control. (B) pHMs or various siRNA pHMs were mock-infected or infected with MV. TNF protein in the culture supernatants was assessed by ELISA 24 h post-infection. (C) IRF7 expression and TNF production kinetics. Control siRNA pHMs or IRF7 siRNA pHMs were infected with MV for various times as indicated and TNF protein in the culture supernatants was assessed by ELISA. (D) IRF7 is required for sustaining TNF gene transcription activated by MV infection. Control siRNA pHMs or IRF7 siRNA pHMs were infected with MV for various times (below lanes) and the cells were analyzed by RT-PCR for the indicated TNF mRNA levels. GAPDH was used as control. Data in (B) and (C) represent mean +/− SD.

IRF7 shares considerable structural and functional similarities with IRF3 [36],[42]; therefore, we investigated whether IRF7 also played a role in RIG-I-mediated TNF induction in MV-infected pHMs. To this end, we first analyzed IRF7 protein levels in MV-infected pHMs by immunoblotting. We found that IRF7 protein was not detectable in uninfected pHMs, but was significantly induced by MV infection (Figure 5A). Notably, MV-induced IRF7 expression in pHMs became markedly augmented at 12–16 h after infection and remained elevated even at 24 h post-infection. To determine whether IRF7 was involved in RIG-I-mediated TNF induction, we silenced MV-induced expression of IRF7 in pHMs using IRF7 siRNA (Figure 5A, middle panel). Next, we measured TNF secretion in the media of control and IRF7-silenced pHMs at 24 h after MV infection. IRF7 silencing reduced MV-elicited TNF production, but to a lesser degree than with IRF3 silencing (Figure 5B). Subsequently, we compared the kinetics of MV-elicited TNF production in pHMs. Initially, the amounts of TNF protein secreted by control and IRF7-silenced pHMs were similar during the first 12 h of MV infection. However, further increases in TNF protein were significantly impaired in IRF7-silenced pHMs at later time points (Figure 5C).

In order to ascertain whether IRF7 affected MV-elicited TNF induction at the transcriptional or post-transcriptional level, we analyzed TNF mRNA in both control and IRF7-silenced pHMs after various times of MV infection. As revealed by RT-PCR, TNF mRNA was induced to similar levels in both groups at the early times (8–12 h) (Figure 5D). In contrast, much less TNF mRNA was detected at the later times in IRF7-silenced pHMs after MV infection (Figure 5D). These results are congruent with the TNF protein data (Figure 5C) and suggest that IRF3 is responsible for the initial triggering of TNF induction while IRF7 impacts the secondary amplification of TNF expression by sustaining TNF gene transcription in MV-infected pHMs.

**Figure 6:**
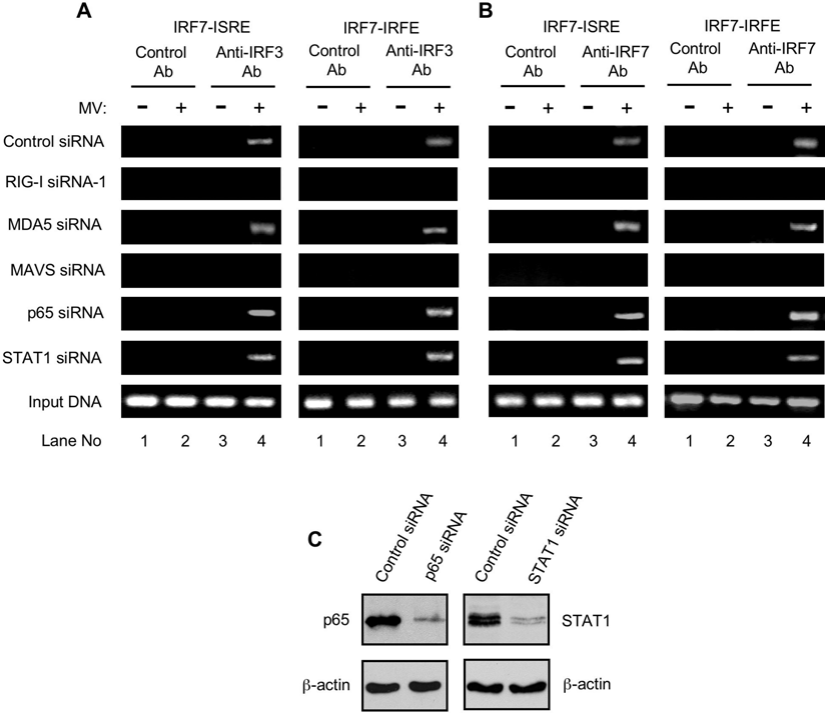
MV induces IRF3 and IRF7 binding to the endogenous IRF7 promoter in primary human macrophages. (A, B) Chromatin immunoprecipitation assay. IRF3 and IRF7 binding to the ISRE and IRFE in the endogenous IRF7 promoter was determined in control siRNA pHMs or various siRNA pHMs as indicated after MV infection for 12 h using PCR to detect IRF7 ISRE and IRFE in immunoprecipitated chromatin fragments. Lanes 1 and 2 are the PCR results for immunoprecipitated samples with the control antibody (Ab). Lanes 3 and 4 show PCR amplification of target sequences in immunoprecipitated chromatin fragments with anti-IRF3 (A) or anti-IRF7 (B) antibody. Input represents PCR amplification of the total input DNA in each sample from the corresponding treatment. (C) pHMs were transfected with control siRNA or specific siRNAs as indicated. The cells were analyzed 72 h later by immunoblotting for protein levels of NF-κB p65 (left) and STAT1 (right). β-actin was used as control.

The prominent role of IRF7 in sustaining MV-elicited later stage TNF production piqued our interest in the primary regulatory pathway involved in MV-induced IRF7 expression. In this regard, previous work shows that virus-activated IRF3 and IRF7 can bind to the IFN-stimulated response element (ISRE) and IRF-binding element (IRFE) in the endogenous IRF7 promoter to initiate IRF7 transcription [43]. Therefore, we performed a chromatin immunoprecipitation (ChIP) assay to determine whether MV infection of pHMs induced IRF3 and IRF7 binding to the IRF7 ISRE and IRFE at 12 h after infection, as IRF7 first became markedly upregulated at this time point (Figure 5A, top panel). We observed that the PCR-amplified DNA bands diagnostic for the ISRE and IRFE elements in the endogenous IRF7 promoter were detected in the anti-IRF3 (Figure 6A, top panel) and anti-IRF7 (Figure 6B, top panel) antibody immunoprecipitates of MV-infected pHMs, but not in the control antibody immunoprecipitates. Because TNF-NF-κB and IFN-STAT1 pathways are known to activate the IRF7 gene [36],[44],[45], we silenced the expression of NF-κB p65 (Figure 6C, left) and STAT1 (Figure 6C, right) to ascertain whether these signaling cascades had any impact on the observed IRF3 and IRF7 binding. Notably, the specific DNA bands were unaffected by silencing of NF-κB p65 (Figures 6A and B, 5th panels) or STAT1 (Figures 6A and B, 6th panels). In particular, STAT1 forms a heterotrimeric complex called ISGF3 (IFN-stimulated gene factor 3) with STAT2 and IRF9 following the activation by type I IFN and is a major stimulator for IRF7 gene expression [36]. Thus, to further define the STAT1 silencing effects, we observed that no positive ChIP signals were detected for IRF7 gene promoter in STAT1 siRNA pHMs following IFN-αA treatment (data not shown). These results together indicate that NF-κB p65 and STAT1 are not involved in MV-induced direct binding of IRF3 and IRF7 to the endogenous IRF7 promoter. Significantly, no specific DNA bands diagnostic for the IRF7 ISRE/IRFE promoter elements were detectable when the expression of RIG-I (Figures 6A and B, 2nd panels) or MAVS (Figures 6A and B, 4th panels) was knocked down as shown in Figure 3A and Figure 4A, respectively. In contrast, when MDA5 was silenced as shown in Figure 3A, ChIP retained the positive signals (Figures 6A and B, 3rd panels). This result is congruent with the findings that MDA5 silencing itself had little inhibitory impact on MV-induced activation of IRF3 and IRF7 (data not shown). Taken collectively, these data suggest that both IRF3 and IRF7 are directly involved in promoting MV-triggered IRF7 gene transcription through RIG-I and MAVS, but independently of type I IFN and NF-κB signaling.

## Discussion

Deciphering the cellular sensing mechanisms involved in virus-elicited TNF or IFN induction is key for understanding the biology of these cytokines in innate antiviral defenses. In this study, we show that MV infection of pHMs triggers the cytoplasmic RNA sensor RIG-I, which activates a MAVS-IRF3/IRF7 cascade leading to the co-induction of TNF and type I IFN. Although IRF3 has been shown to mediate TNF induction under various circumstances, such as lipopolysaccharide stimulation or certain viral infections [38]–[41], the collaboration between IRF3 and IRF7 resulting in sustained TNF induction has not been recognized before. Thus, our study has revealed novel insights into the signaling control of TNF production, and its close linkage with type I IFN upregulations in virus-infected human macrophages.

The first indication of potential involvement of the cytoplasmic RNA helicases in sensing DNA virus infection came from the knockdown of MAVS (also called IPS-1), the sole known adaptor for RIG-I and MDA5 signaling, in which IFN-β induction was shown to be substantially reduced in MAVS-deficient mouse embryo fibroblasts following the infection by modified vaccinia Ankara [46]. More recently, DNA viruses herpes simplex virus-1 and adenovirus were shown to replicate to much higher titers in RIG-I mutant human hepatoma cells Huh-7.5.1. than in the parental Huh-7 cells [47]. The critical function of RIG-I in sensing MV infection of pHMs raises an interesting question about why MDA5 has an apparently less significant role in triggering MV-elicited induction of TNF and IFN-β. Although RIG-I and MDA5 belong to the same family of DexD/H box helicases [9], MDA5 is known to preferentially recognize picornaviruses and poly(I∶C) [13]. Thus far, MDA5 deficiency has been shown to have either no effect, or only a slight inhibitory impact, on the type I IFN responses elicited by other RNA and DNA viruses [48]. Taken together with these previous studies, our data thus point to an emerging theme that RIG-I may play a more general role in innate recognition of various DNA viruses.

This is of importance considering the fact that information remains limited about the cytoplasmic DNA sensor(s). Presently, the only characterized cytoplasmic DNA sensor DAI, also known as DLM-1/ZBP1, was shown to mediate type I IFN induction following infection with herpes simplex virus-1 in L929 cells [49]. However, sensing of cytoplasmic DNA by DAI was found to be dispensable in mouse embryo fibroblasts [50]. Thus, it appears that the role of DAI in DNA sensing may be a cell type specific or functional status-related phenomenon. In the present study, we observed that DAI knockdown inflicted only a minor inhibitory effect on TNF and IFN-β induction in MV-infected pHMs (data not shown). This result seems to further emphasize the importance of RIG-I as an innate sensor for DNA virus infections.

Our previous study of MV tropism restriction showed that MV infection not only induced type I IFN in primary mouse embryo fibroblasts but MV replication was exquisitely sensitive to the induced IFN, demonstrating that the type I IFN response is a predominant tropism determinant for MV in mouse fibroblasts [3]. Of note, a previous study using primary human neonatal foreskin fibroblasts reported that the early passage (P1–3) fibroblasts were permissive for MV infection, while late passage cells (P5–10) became resistant at a time that paralleled the acquisition of constitutive IFN-β secretion, thus indicating a major role for type I IFN in inhibiting MV infection in those cells [51]. In the present study, however, we found that adult breast skin fibroblasts (CCD-922Sk), adult abdomen skin, fetal lung and fetal conjunctival fibroblasts uniformly supported permissive MV infection, regardless of the passage status, and all required both TNF and type I IFN to fully inhibit MV proliferation (data not shown). Taken together, these findings suggest that both TNF and type I IFN are necessary for a full-fledged inhibition of MV infection in a wide variety of the primary human fibroblasts tested although type I IFN itself might still be able to act as a potent inhibitor in certain cells.

We also report here that MV infection co-induced TNF and type I IFN only in pHMs, but not in primary fibroblasts or lymphocytes, in a RIG-I-dependent fashion. Hence, these data imply either that certain MV gene products act as RIG-I signaling pathway inhibitor(s) only in fibroblasts and lymphocytes or else there exist unique signaling elements of the RIG-I pathway that are restricted to pHMs. In either event, MV immunosubversion effectors may well be inherently involved in the determination of MV tropisms in primary human cells in a cell type-dependent manner.

Although disruption of RIG-I signaling is conducive for MV replication in pHMs, high titers of MV progeny are not achieved because of premature death of MV-infected pHMs (data not shown). This appears to lend support to an emerging notion that the major function of pHMs in combating MV infection is to provide an altruistic source of TNF and type I IFN. It has been known for over a half-century that humans are highly resistant to MV infection, even following direct injections of live MV [1], but the molecular basis underlying the innate cellular restriction to MV infection in humans has never been elucidated. Thus, this novel RIG-I-coordinated signaling of TNF and type I IFN in leukocytes such as pHMs would provide some clues to explain how MV infection might be restricted in humans *in vivo*. In this regard, future clinical trials of MV as a new oncolytic virus to treat human cancers may provide a unique opportunity to determine the actual physiological relevance of leukocyte-based TNF and type I IFN responses in humans at the whole organism level.

Like IFN, TNF can also cause significant harm to the host when over-expressed [52]. Thus, a tight control over both TNF and type I IFN induction and shut-off is a key to homeostasis. Here, we show that IRF3 activation alone triggered only a transient upregulation of TNF and type I IFN. In most cell types, IRF3 is constitutively expressed, whereas IRF7 must usually be induced *de novo* [36],[37]. Thus, the sequential involvement of IRF3 and IRF7 in the early and later phase of TNF production allows us to postulate that, if the early phase production of TNF and type I IFN is sufficient to block viral infection, then the later phase production of both cytokines will be coordinately curtailed. Taken collectively, sustaining TNF induction through IRF3 and IRF7 thus provides the host with a fine-tuned regulatory mechanism to allow for only needed TNF expression in parallel with type I IFN. In particular, the identification of IRF7 as a key regulator for sustained production of both TNF and type I IFN may offer useful clues for designing new therapeutic strategies for disorders in which over-expression of either cytokine is suspected as a major culprit.

Of relevance, recent work shows that in lipopolysaccharide-stimulated mouse macrophages, TLR4 interacts initially with MyD88 to mediate the early phase TNF expression and then with the newly induced 4-1BBL to sustain longer term TNF production [53]. Thus, sustained induction of TNF may be an intrinsic feature that can take place through distinct signaling pathways in a stimulus-specific fashion.

A pivotal step in sustaining the MV-elicited co-induction of TNF and type I IFN in pHMs is the *de novo* synthesis of IRF7. Our ChIP data reveal that MV-stimulated MAVS signaling caused the direct binding of both IRF3 and IRF7 to the ISRE and IRFE sites in the IRF7 promoter, and that this is unaffected by STAT1 or NF-κB p65 knockdown. Notably, previous studies show that type I IFN and TNF each potently stimulate IRF7 expression [36],[44],[45]. In this study, we observed that, in addition to TNF, MV infection co-induced type I IFN in pHMs. We further observed that IRF7 was strongly induced in pHMs by either exogenous type I IFN or TNF (data not shown), thus indicating that MV-elicited type I IFN and TNF also likely contribute to IRF7 upregulation in pHMs. Taken together, these data suggest that IFN-STAT1, TNF-NF-κB and MAVS-IRF3/IRF7 signaling cascades are mobilized independently following MV infection to induce IRF7 expression in pHMs. Because the protein synthesis of both type I IFN and TNF requires that IRF3/IRF7 be activated first, the direct binding of virally activated IRF3 and IRF7 to the ISRE and IRFE in the IRF7 promoter thus provides a rapid shortcut to trigger IRF7 transcription, whereby an initial weak signal serves as the positive feedforward/feedback platform for a strong IRF7 expression at the later times through robust secondary response pathways of IFN-STAT1 and TNF-NF-κB.

In summary, our study shows that RIG-I sensing of a DNA poxvirus infection in pHMs is critically linked to co-regulated TNF and type I IFN responses that control viral tropism in primary human cells in general. These data indicate that cytoplasmic RNA sensors may play a more important role than previously envisaged in triggering the innate antiviral cellular defenses against DNA virus infections in human cells.

## Materials and Methods

### Human fibroblasts, macrophages and lymphocytes

Primary human skin fibroblasts CCD-922Sk (kindly provided by Dr. Scot Roberts) were maintained in MEM with 10% FBS, penicillin (100 u/ml) and streptomycin (100 µg/ml). To prepare pHMs and primary human lymphocytes, human peripheral blood mononuclear cells were first obtained by density gradient centrifugation over HISTOPAQUE-1077 (Sigma) according to the product instruction. Subsequently, the isolated peripheral blood mononuclear cells were suspended in RPMI 1640 supplemented with 5% FBS, penicillin (100 u/ml) and streptomycin (100 µg/ml) and were allowed to adhere to plastic plates for 1 h. The adherent monocytes were then separated from the nonadherent lymphocytes by repeated washing with plain medium. The monocyte-depleted nonadherent leukocyte fractions were collected as the primary human lymphocytes for MV infection experiments whereas the adherent mononcytes were cultured in RPMI 1640 supplemented with 10% autologous human serum for 7 days to allow differentiation into macrophages as described previously [54].

### MV and recombinant MV-hTNF

MV (Lausanne strain) with *lacZ* gene inserted at an innocuous intergenic site under the control of a late viral promoter was described previously [55]. UV-inactivated MV was prepared with a Stratagene Stratalinker. To construct MV-hTNF, using vMyxlac as the parental virus, we first conducted PCR synthesis of flanking sequences for homologous recombination. The left flanking sequence (FL) for insertion into MV ORF M131R, a nonessential gene for MV replication, was synthesized with primer pair: FLF_KpnI: *GGTACC* ATACGACGTCGTACGCGAA TCTG GCA and FLR_MCS (multi-cloning site): *AAGATCTAAGTCGACCCCGGG* *TAATGAA TTCTACGTAGCGGCCGCAAGCTAGC* TCTTTATTAAACTCGTA ATAGCGAGGA. The right flanking sequence (FR) was made similarly with the primer pair: FRR_SacI: *GAGCTC* TAGTATACAGCGATACGTCAATGGACA and FRF_MCS: *AGCTAGCTTGCGGCCGCTACGTAGAATTCATTACCCGGGG* *TCGACTTAGATCT* AACCGGTGATCGATCCATTATAGGA. Note that the above-italicized letters indicate MCS. Then, the FL and FR PCR products were mixed and ligated with a second round PCR using the primer pair FLF_KpnI and FRR_SacI. The flanking sequence containing the MCS was cloned into pBluescriptKS+ between KpnI and SacI, and the sequence fidelity was confirmed. The MCS contains RE sites: NheI, NotI, EcoRI, SmaI, SalI, and BglII. Next, the *gpt* gene driven by vaccinia virus early/late promoter p7.5 was inserted into the BglII site and *EGFP* driven by a synthetic poxvirus early/late promoter [56] or *EGFP* and human *TNF*, each controlled independently but in the opposite directions by synthetic poxvirus early/late promoters, were inserted between NheI and SmaI sites. Standard protocols for making recombination poxviruses were used [57]. The titration of MV-hTNF progeny virus was conducted on RK-13 using the standard plaque assay as described [55]. For confirmation of TNF expression by MV-hTNF, the whole cell lysates and supernatants from the RK-13 cells infected by either control MV or MV-hTNF were rendered for immunoblotting analysis using an anti-human TNF antibody.

### Virus infection, X-gal staining, β-gal and viral yield assays

MV was used at an MOI of 0.0l for X-gal staining and β-gal assay and at an MOI of 1.0 for RT-PCR, immunoblotting, ELISA and ChIP experiments. X-gal staining with 5-bromo-4-chloro-3-indolyl-β-D-galactopyranoside (Sigma) was performed to visualize MV blue foci 48 h after infection as described [3]. Microscopic images of X-gal staining were taken with a Leica DMIRE2 microscope. MV β-gal assay was performed as previously described [58] with *o*-nitrophenyl-β-D-galactopyranoside (Sigma) 48 h after infection. The β-gal activity was normalized as optical density at 420 nm per milligram of total cellular protein. For viral yield assays at 48 h after infection, titers of EMCV infection samples were determined on BHK cells [59] while MV titers were determined on BGMK cells [55] in terms of plaque-forming units per ml (pfu/ml) using the standard plaque assay.

### Cytokine treatments, primary antibodies, immunoblotting, ELISA and neutralization experiments

Recombinant human IFN-β and TNF were from PBL Biomedical Laboratories and Biosource International, respectively. IFN-β was used at 150 units/ml while TNF was used at 10 ng/ml. Two cytokines were either added alone or together to the media when the infection with MV or EMCV was started and maintained throughout the entire treatment period. The primary antibodies were obtained from the following suppliers: anti-IRF3, anti-IRF7, anti-NF-κB p65 and anti-USF2 antibodies, Santa Cruz Biotechnology; anti-human TNF and anti-human IFN-α/β antibodies, Biosource International; anti-β-actin antibody, Sigma; anti-STAT1 antibody, Transduction Laboratories. For immunoblotting, whole cell extracts were prepared at each specified time after various treatments as previously described [3]. The preparation of nuclear extracts was done according to the published procedures [60]. The extracted proteins were resolved on 7.5–12% SDS-PAGE and transferred to Hybond-C nitrocellulose (Amersham Pharmacia Biotech). Immunoblotting was performed with respective primary antibodies, and bands were visualized with secondary HRP-conjugated antibodies and ECL system (NEN Life Science Products). To quantitate TNF and IFN-β, the supernatants from MV-infected cell cultures were collected at various time points as specified after treatments and subjected to analysis by standard sandwich ELISA as described [61]. For the neutralization of TNF or IFN-α/β, or both TNF and IFN-α/β, anti-human TNF antibody (20 µg/ml), or anti-human IFN-α/β antibodies (each at 1,000 neutralization units/ml), or both anti-human TNF and anti-human IFN-α/β antibodies were added, respectively, to the medium 2 h prior to MV infection of pHMs as indicated and kept throughout the entire infection period.

### Transfection of siRNAs and poly(I∶C)

The siRNA oligonucleotides targeting human RIG-I (NM_014314), MDA5 (NM_022168), MAVS (VISA, NM_020746), IRF3 (NM_001571) and IRF7 (NM_001572) as well as control siRNA siCONTROL #1 were obtained from DHARMACON. The two individual pairs of RIG-I siRNA sequences are as follows: RIG-I siRNA-1: sense, CAGAAGAUCUUGAGGAUAAUU, antisense, 5′-P.UUAUCCUCAAGAUCUUCUGUU), and RIG-I siRNA-2: sense, GCACAGAAGUGUAUAUUGGUU, antisense, 5′-P.CCAAUAUACACUUCUGUGCUU. Human NF-κB p65 and STAT1 siRNAs were obtained from Cell Signaling Technology. siRNAs were transfected into pHMs using HiPerFect Transfection Reagent (Qiagen) according to the manufacturer's specifications. At 48 or 72 h after the transfection of various siRNAs as indicated, the cells were used for further experiments. Synthetic dsRNA polyinosinic-polycytidylic (poly(I∶C)) was from Sigma. For transfection, cells were incubated for various times as indicated with 1.5 µg/ml of poly(I∶C) complexed with Lipofectamine 2000 (Invitrogen) according to the manufacturer's instructions.

### Immunofluorescence microscopy

For immunofluorescent staining, pHMs were grown on coverslips. After infection with MV for the indicated times, the infected cells were fixed in cold methanol for 10 min, washed in PBS and blocked in 5% normal goat serum for 50 min. A rabbit anti-IRF3 antibody was then applied at room temperature for 1 h followed by a 45-min secondary detection with Texas Red-conjugated goat anti-rabbit IgG. Thereafter, the nuclear DNA of the immunolabeled cells were counterstained with DAPI (4′,6-diamidino-2-phenylindole, dihydrochloride, Molecular Probes) according to the product specification. The secondary antibody conjugate was from Jackson ImmunoResearch Laboratories. The fluorescence images were taken with a Leica DMIRE2 microscope.

### RT-PCR and chromatin immunoprecipitation assay

After various treatments as indicated, total RNA was isolated using a RNeasy kit (Qiagen). Reverse transcription was performed using Superscript reverse transcriptase (Invitrogen). RT-PCR conditions and primer sequences for human TNF and GAPDH [62], IFN-β [63], RIG-I, MDA5 and MAVS [64] were described.

ChIP assay was conducted as described previously [43]. Briefly, pHMs were infected with MV for 12 h and then subjected to cross-linking by formaldehyde. Immunoprecipitation was performed with the specific antibodies or a control antibody (rabbit IgG) as indicated. After reversion of the protein-DNA cross-links in the immunopreciptitates, the purified DNA was analyzed by PCR for the sequences that contain ISRE and IRFE in the endogenous IRF7 promoter. PCR conditions and the specific primers for IRF7 ISRE and IRFE were described [43].

## Supporting Information

Figure S1 Confirmation of insertion and expression of human TNF gene in MV genome. (A) Control MV DNA or recombinant MV-hTNF DNA was extracted and analyzed with PCR pairs as specified in Materials and Methods. Lanes 1 and 2: control MV. Lanes 3 and 4: MV-hTNF. (B) RK-13 cells were infected by either control MV or MV-hTNF as indicated for 24 h. The whole cell lysates and supernatants were collected and rendered for immunoblotting analysis using an anti-human TNF antibody.
(0.90 MB TIF)

Figure S2 Two pairs of RIG-I siRNA oligos share similar silencing phenotypes in primary human macrophages. (A) pHMs were transfected with control siRNA or RIG-I siRNA-1 or RIG-I siRNA-2 as indicated (top panel). The cells were analyzed 48 h later by RT-PCR for RIG-I mRNA levels. GAPDH was used as control. (B, C) pHMs or various siRNA pHMs as indicated were mock-infected or infected with MV for 24 h and the accumulation of TNF and IFN-β in the culture supernatants was assessed by ELISA. Data in (B) and (C) represent mean +/− SD.
(0.77 MB TIF)

Figure S3 RIG-I siRNA transfection does not activate nonspecific type I IFN response in pHMs. (A) pHMs were mock transfected or transfected with poly(I∶C) or with RIG-I siRNA-1 or RIG-I siRNA-2 for 24 h. IFN-β accumulation in the culture supernatants was assessed by ELISA. Data represent mean +/− SD. (B) pHMs were mock transfected or transfected with poly(I∶C) or with RIG-I siRNA-1 or RIG-I siRNA-2 for 12 h. Total RNA was analyzed by RT-PCR for induction of IFN-β mRNA. GAPDH was used as control.
(0.70 MB TIF)
