# Supplementary figures and images for: RIG-I Mediates the Co-Induction of Tumor Necrosis Factor and Type I Interferon Elicited by Myxoma Virus in Primary Human Macrophages
Source: PLoS Pathog. 2008 Jul 11;4(7):e1000099. doi: 10.1371/journal.ppat.1000099 (PMC2438611; doi:10.1371/journal.ppat.1000099)

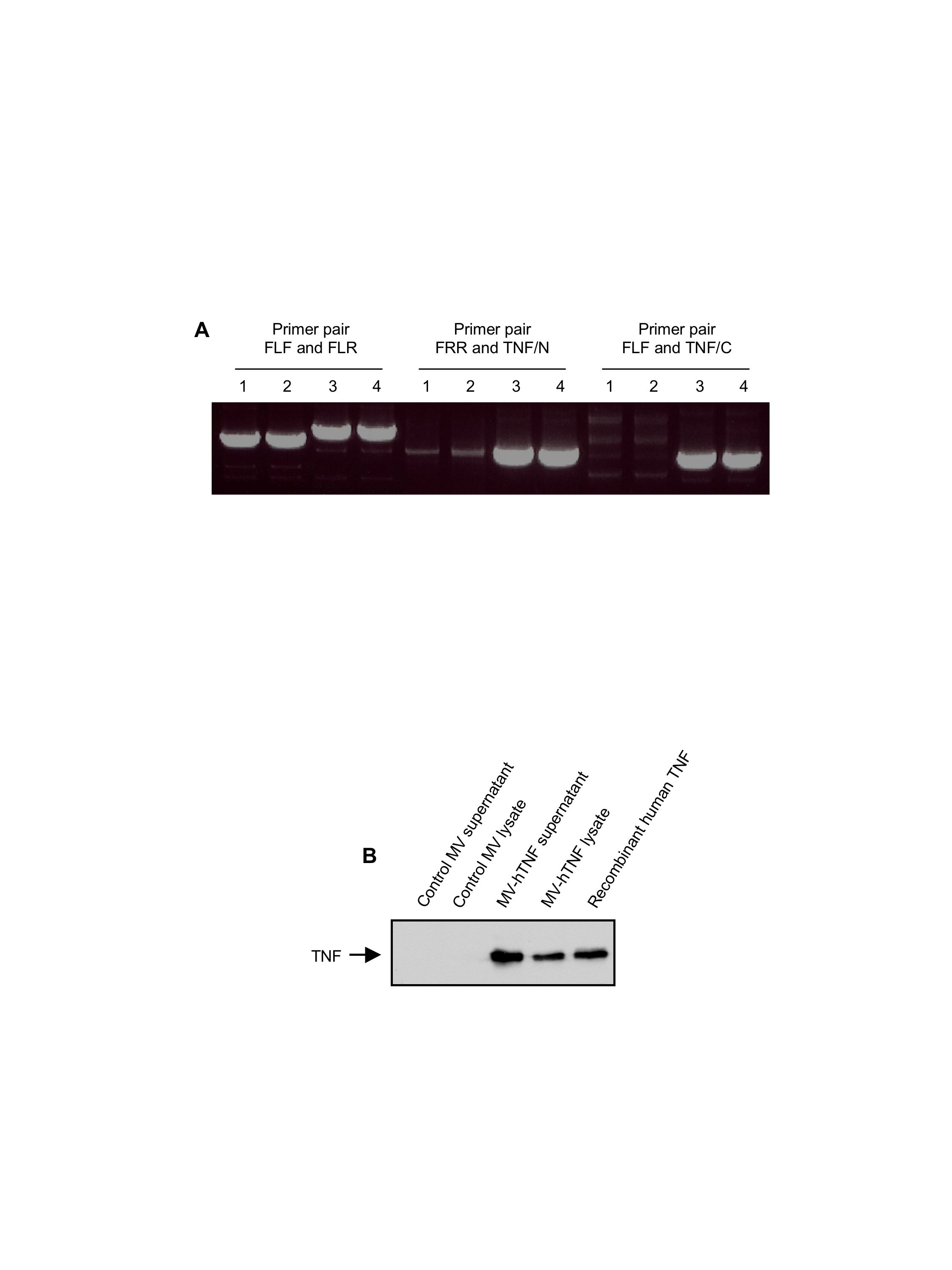

Supplement: Figure S1 — Confirmation of insertion and expression of human TNF gene in MV genome. (A) Control MV DNA or recombinant MV-hTNF DNA was extracted and analyzed with PCR pairs as specified in Materials and Methods. Lanes 1 and 2: control MV. Lanes 3 and 4: MV-hTNF. (B) RK-13 cells were infected by either control MV or MV-hTNF as indicated for 24 h. The whole cell lysates and supernatants were collected and rendered for immunoblotting analysis using an anti-human TNF antibody. (0.90 MB TIF) [file ppat.1000099.s001.tif]

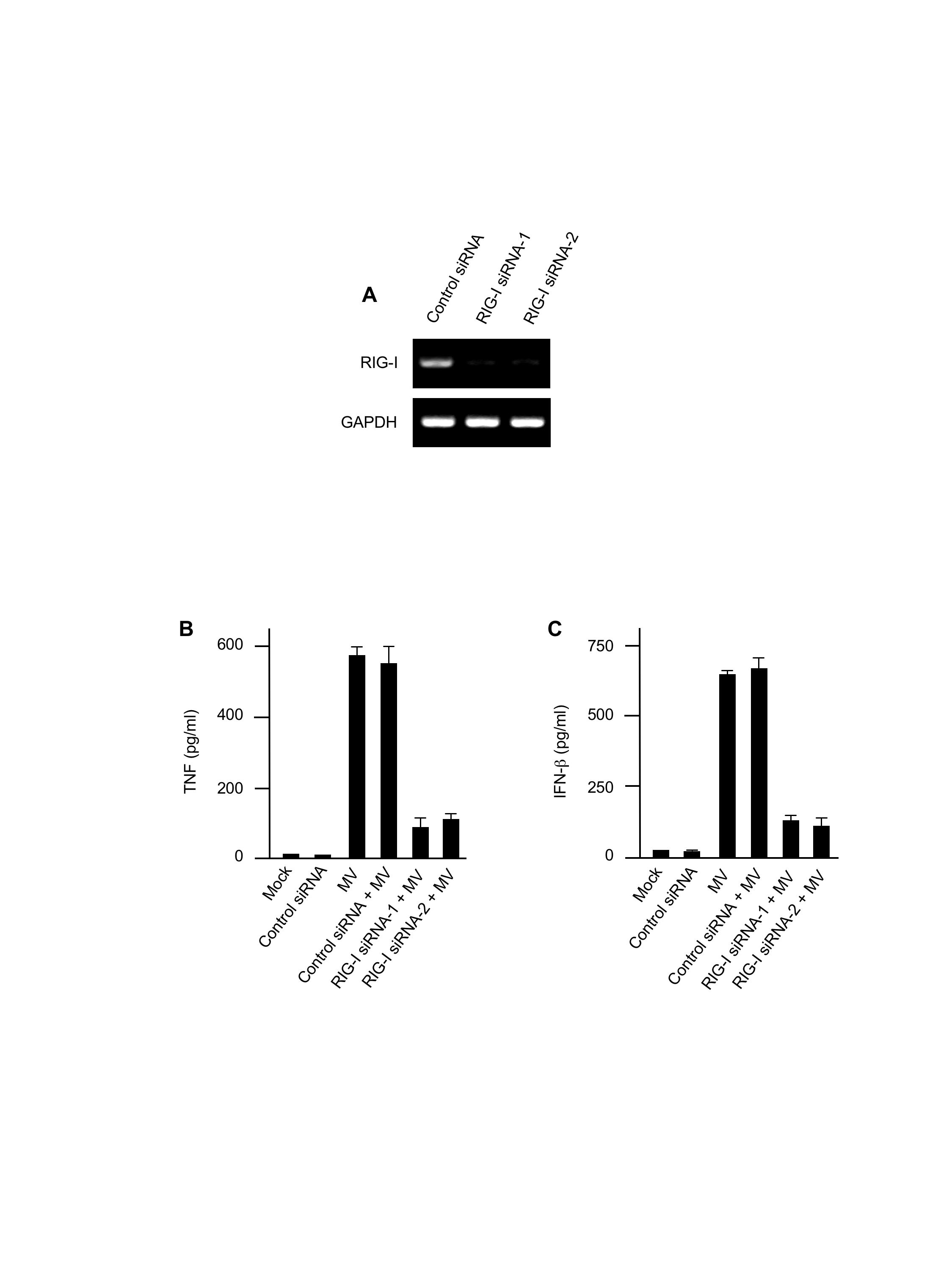

Supplement: Figure S2 — Two pairs of RIG-I siRNA oligos share similar silencing phenotypes in primary human macrophages. (A) pHMs were transfected with control siRNA or RIG-I siRNA-1 or RIG-I siRNA-2 as indicated (top panel). The cells were analyzed 48 h later by RT-PCR for RIG-I mRNA levels. GAPDH was used as control. (B, C) pHMs or various siRNA pHMs as indicated were mock-infected or infected with MV for 24 h and the accumulation of TNF and IFN-β in the culture supernatants was assessed by ELISA. Data in (B) and (C) represent mean +/− SD. (0.77 MB TIF) [file ppat.1000099.s002.tif]

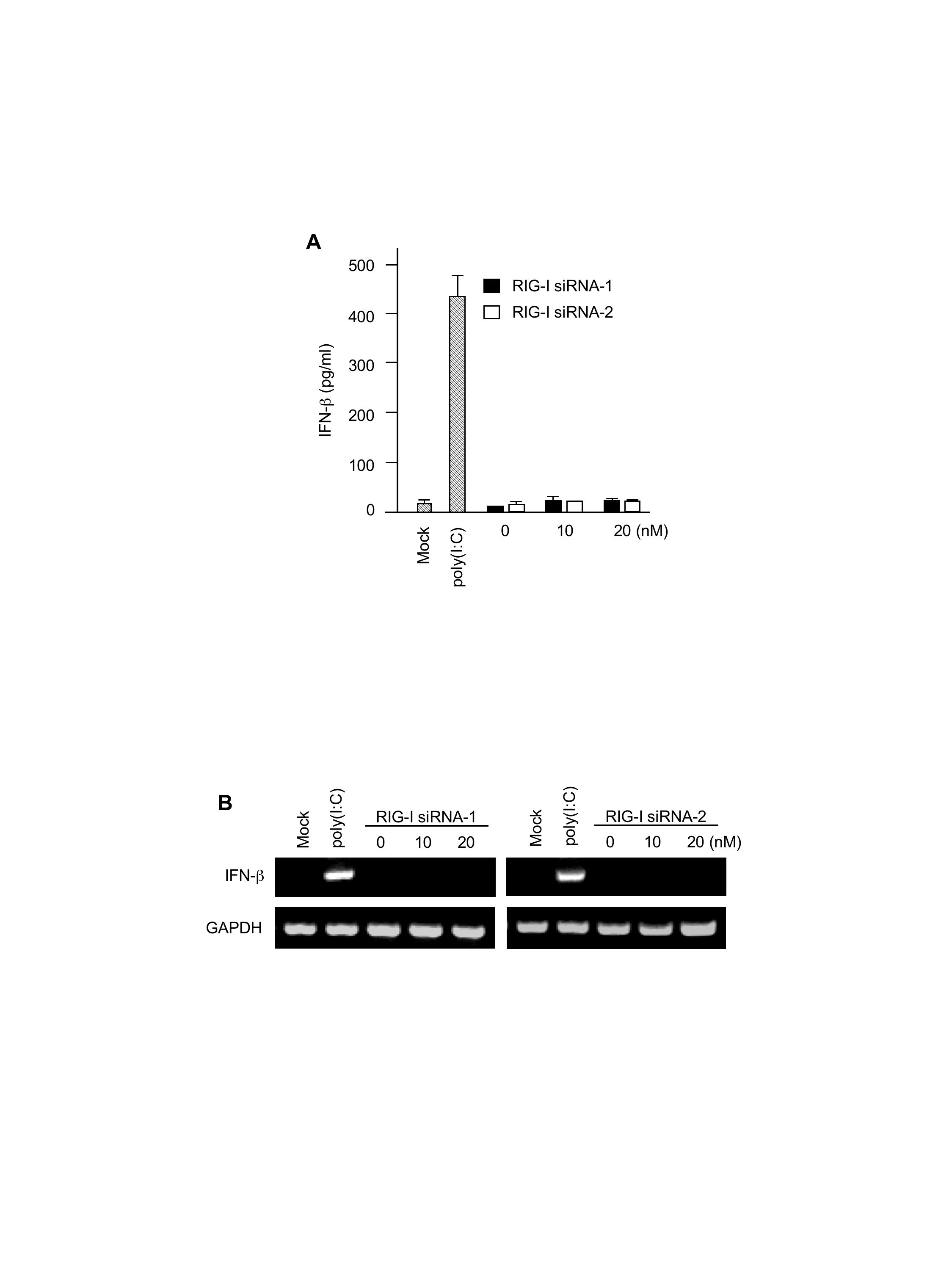

Supplement: Figure S3 — RIG-I siRNA transfection does not activate nonspecific type I IFN response in pHMs. (A) pHMs were mock transfected or transfected with poly(I∶C) or with RIG-I siRNA-1 or RIG-I siRNA-2 for 24 h. IFN-β accumulation in the culture supernatants was assessed by ELISA. Data represent mean +/− SD. (B) pHMs were mock transfected or transfected with poly(I∶C) or with RIG-I siRNA-1 or RIG-I siRNA-2 for 12 h. Total RNA was analyzed by RT-PCR for induction of IFN-β mRNA. GAPDH was used as control. (0.70 MB TIF) [file ppat.1000099.s003.tif]
